# Midline mandibulotomy for large carotid body tumors: a valuable approach

**DOI:** 10.1016/j.jvscit.2022.11.010

**Published:** 2023-03-02

**Authors:** Ahmed Almumtin, Mohammed Dahman, Anas Alkhudari, Maad Galal, Albeshr Almasri, Samer Koussayer

**Affiliations:** a Department of Surgery, King Faisal Specialist Hospital and Research Center, Riyadh, Kingdom of Saudi Arabia; b Alfaisal University, Riyadh, Kingdom of Saudi Arabia

**Keywords:** Carotid body tumor, Carotid space, Midline mandibulotomy, Neck tumor, Paraganglioma

## Abstract

In the present case, a 41-year-old male patient had reported a large swelling on the right side of his neck that had been radiographically diagnosed as a carotid body tumor. The tumor extended toward the right thyroid lobe, deep to the parapharyngeal space and cranially to the base of the skull. The clinical and medical imaging findings confirmed the diagnosis. Because of the large size of the tumor (50 mm × 48 mm × 85 mm), extent of involvement, and hypervascularity, surgical excision by midline mandibulotomy was chosen as the treatment approach. Midline mandibulotomy is a very good approach for large tumors with extensive involvement of the surrounding tissues, especially tumors with great medial and cephalic extension.

Carotid body tumors (CBTs), also known as paragangliomas or chemodectomas, are slow-growing tumors originating from the chemoreceptor cells and are more prevalent among middle-age women.^1^ CBTs usually arise near the carotid bifurcation within glomus cells derived from the embryonic neural crest cells. They are rare tumors with an incidence of one to two cases per 100,000 persons, accounting for 60% of head and neck paragangliomas.^2^

The most frequently noted CBT symptoms are discomfort, dysphagia, and autonomic dysfunction.^1^ The radiographic tools useful for the CBT diagnosis include computed tomography angiography (CTA), magnetic resonance angiography, and ultrasound.^3^ A recent classification was compared with the classification by Shamblin and adopted by the Peking Union Medical College Hospital (PUMCH). In the PUMCH classification, vertical extension was found to correlate well with the clinical outcomes. Using the PUMCH classification, our tumor was type 5 (ie, the tumor extended proximally to the mastoid process tip and had engulfed both carotid vessels).^4^ For large tumors that extensively involve the adjacent carotid artery and those extending to the parapharyngeal space and/or skull base, the midline mandibulotomy approach is a valuable method because it provides full anatomic access.^5^^,^^6^

In the present report, we have described the case of a patient with a Shamblin type III, PUMCH type 5, 50 mm × 48 mm × 85 mm CBT. Such a large size and extension make surgical resection very critical but could render the tumor inoperable. Therefore, the midline mandibulotomy approach allows for better anatomic visibility and better dissection. The patient provided written informed consent to report his case details and imaging studies.

## Case report

**Fig 1:**
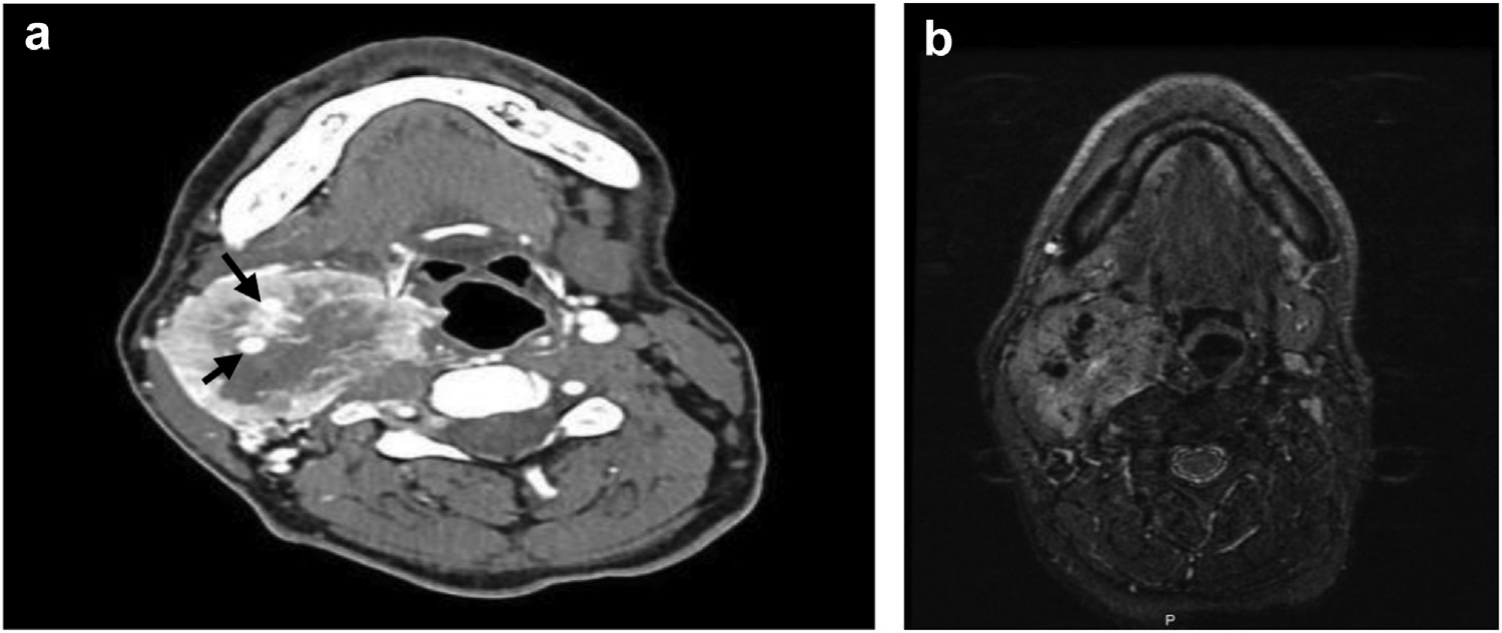
**A,** Computed tomography angiography (CTA) showing large carotid body tumor (CBT) with encasement of external and internal carotid arteries (ECA and ICA, respectively; *black arrows*). **B,** Magnetic resonance imaging (MRI) scan showing large carotid body tumor (CBT) encasing the ECA and ICA.

**Fig 2:**
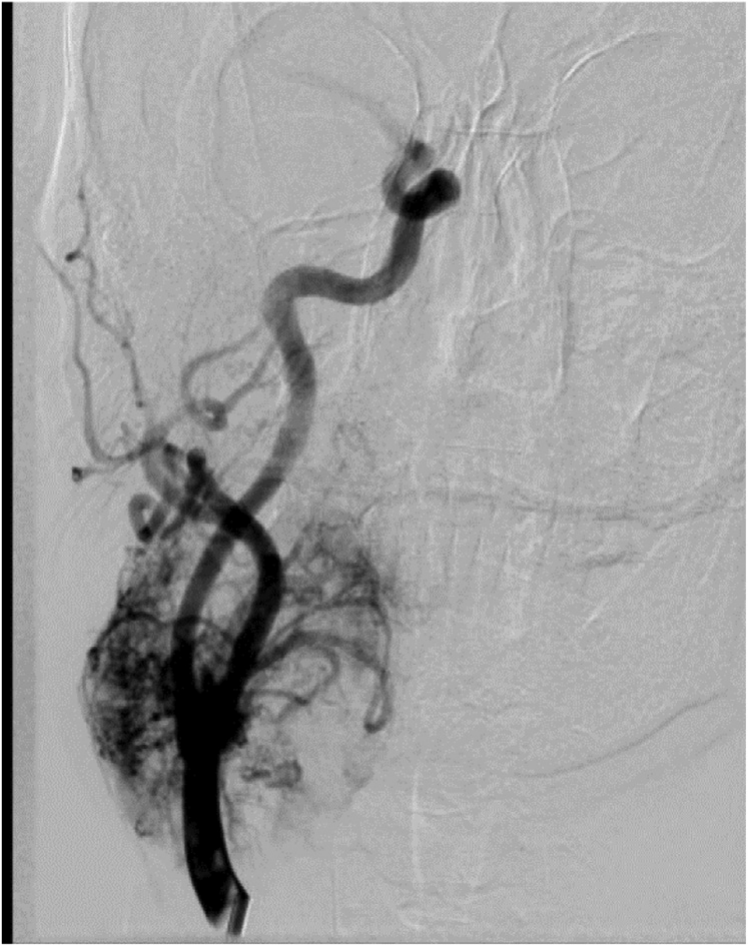
Angiogram showing splaying of the internal and external carotid arteries (ICA and ECA, respectively).

Our patient was a 41-year-old man who was a chronic smoker and known to have ischemic heart disease, peripheral vascular disease, hypertension, and dyslipidemia. He was taking multiple medications, including dual antiplatelet drugs (Aspirin 81 mg, and clopidogril 75 mg orally once daily), a statin (Atorvastatin 40 mg orally once daily), amlodipine (10 mg orally once daily), and ramipril (5 mg orally once daily). He had presented with a right-sided neck mass that had progressively increased in size. The patient complained of a chronic cough and significant weight loss of 13 kg within the 3 months before his presentation. He had no history of dysphagia. On physical examination, the mass was approximately 10 × 10 cm in size, nontender, hard, and immobile. It was fixed to underlying tissues on the right side of his neck without obvious skin changes. The laboratory tests showed no abnormal changes in his catecholamine derivative levels. At presentation, CTA of the neck revealed a large right CBT with enlargement of the internal carotid artery (ICA) and external carotid artery (ECA) and a central area of necrosis. The mass extended caudally to the level of the right thyroid lobe and cranially to the base of the skull. Significant dilatation of the right ECA had occurred to supply the tumor (Figs 1 and 2).

Magnetic resonance imaging (MRI) revealed that the mass was hypervascular, centered in the right carotid space, and measured 50 × 48 × 85 mm in the anteroposterior, width, and craniocaudal dimensions, respectively. The gadolinium-enhanced images revealed intense enhancement with central areas devoid of enhancement because of necrosis. Obliteration of the parapharyngeal space medially and displacement of the sternocleidomastoid muscle anterolaterally were observed. Positron emission tomography showed evidence of a neoplastic process on the right side of the neck with worrisome pulmonary nodules and indeterminate focal uptake of the right transverse process of T4.

**Fig 3:**
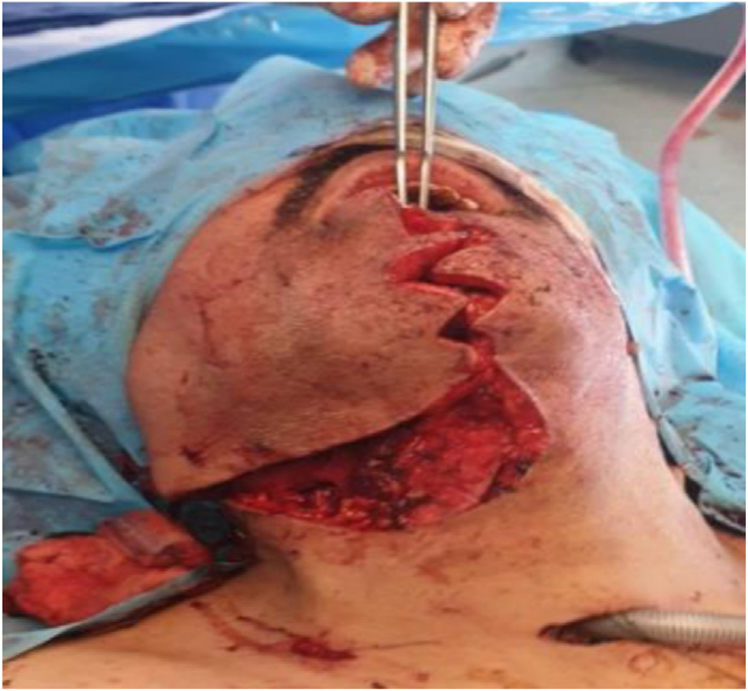
Surgical incision in the mandible and neck via the midline mandibulotomy approach.

A 30-minute balloon test occlusion (BTO) performed under fluoroscopic guidance was negative. The BTO was conducted in an attempt to predict the patient’s ischemic tolerance if the ICA was ligated. In the same setting and after the BTO, preoperative embolization to include multiple feeding vessels was performed and resulted in satisfactory diminution of the angiographic blush (Fig 3).

After a multidisciplinary meeting with the relevant specialties, surgical resection using a midline mandibulotomy approach was decided. The patient was referred for a vocal cord mobility assessment, which revealed normal findings. The patient provided written informed consent for the planned procedure, anticipated complications, other options of management, and the possibility of further treatment. He was advised to stop clopidogrel (Plavix) 4 days preoperatively, although he was instructed to continue aspirin.

### Surgery

**Fig 4:**
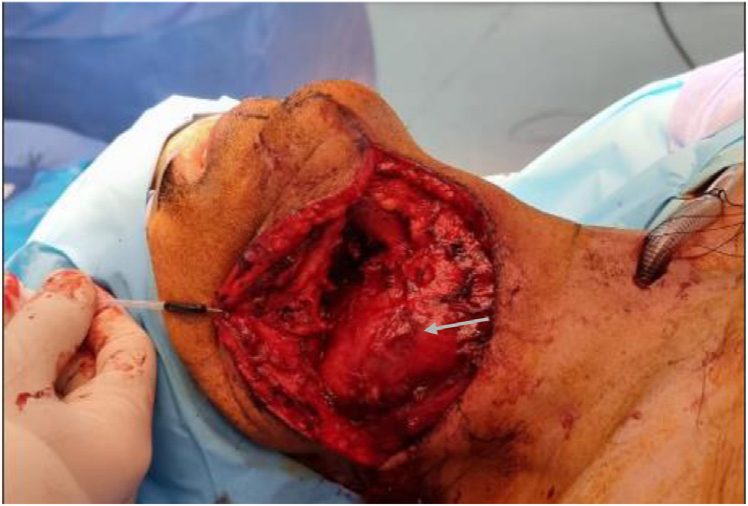
Surgical excision through exposure in the neck (*White arrow* indicates the tumor).

Nasal intubation and tracheostomy were performed by the ear, nose, and throat team owing to the possibility of unilateral cord paralysis, risk of postoperative aspiration, expected laryngeal edema, and a potentially difficult extubation. Subsequently, a midline incision was made in the mid-portion of the lower lip. The incision was extended to the right neck, posteriorly to the base of the ear, and the muscles were cut, including the superior belly of the omohyoid and mylohyoid muscle (Fig 4). A midline mandibulotomy was performed by the maxillofacial surgeon after placing a plate and eight screws to hold them in place after splitting the mandible. On exposure, we found the tumor had engulfed the distal right common carotid artery, ECA, and almost the whole length of the ICA (a 1-cm stump was resected and used for the distal end anastomosis) up to the base of the skull. The tumor had completely encased the vagus nerve (MRI did not show clear involvement) and had invaded the hypoglossal nerve, sympathetic chain, and surrounding muscles. It was completely resected, with the vagus nerve and hypoglossal nerves sacrificed. An interposition reversed saphenous vein graft was used to reconstruct the resected ICA, and the ECA was ligated. Two silicone flat Jackson-Pratt drains were placed. The estimated blood loss was ∼600 mL, and the patient received 2 U of packed red blood cells, each containing 350 mL. The histopathologic examination revealed a malignant-appearing CBT with central necrosis and high mitotic activity, with capsular, perineural, and angioinvasion.

### Postoperative course

Clopidogrel was started again through the nasogastric tube on the second day postoperatively. The first Jackson-Pratt drain was removed on the second day, and the second drain was removed on the fourth day postoperatively when the drain output was <25 mL. At 2 days postoperatively, the patient was found to have right eyelid drooping and an unequal pupil size, which prompted a comprehensive neurological assessment with imaging studies. The presence of Horner syndrome was confirmed, and the cerebral CTA findings were suggestive of a right front parietal stroke, which might have occurred secondary to the thrombosis of the venous conduit despite the negative BTO results.

The patient was transitioned from nasogastric tube feedings to gastrostomy tube feedings because of worsening dysphagia to liquids and solids and a high risk of aspiration, evidenced by two failed swallowing tests at days postoperatively. He underwent physical therapy, occupational rehabilitation, and swallow training. After 4 months of follow-up, his clinical evaluations, CT scans, and MRI scans showed no signs of local recurrence. His swallowing had gradually improved. The patient was referred to radiation oncology for radiation therapy postoperatively.

## Discussion

In the present report, we have described the case of a male patient aged 41 years, an age similar to that reported in previous investigations by Dorobisz et al^7^ and Gad et al.^8^ CTA is the reference standard for diagnosing CBTs. Early surgical resection of CBTs has been recommended to prevent local invasion and potential metastasis; however, when the tumor position is high, the tumor is highly vascular, and extensive surrounding tissue involvement is present, with the increased possibility of morbidity and mortality, surgery can be challenging.

Several approaches have been used for the resection of oropharyngeal and parapharyngeal space tumors and carotid paragangliomas, including the transcervical, transparotid, transoral, and transmandibular approaches, alone or combined. The transmandibular approach or the mandibular swing/split approach has been preferred for tumors that extensively involve the parapharyngeal space, those with significant cephalad extension, those involving the internal jugular vein or carotid artery, large size tumors, and recurrent tumors.^9^ Several methods are available to perform a mandibulotomy, including via the body (lateral mandibulotomy), the midline, and the paramedian.^10^ The midline and paramedian line mandibulotomies with lateral dislocation of the ipsilateral jaw provide excellent visibility of the oral cavity and oropharynx.^4^

In the present case, because of the tumor size, considerable involvement of the surrounding structures, and hypervascularity, a midline mandibulotomy approach was planned. The approach had been previously used by Shankar et al,^11^ and no complications were observed.

Surgical excision of CBTs is an effective and relatively safe procedure but with well-documented rates of serious neurovascular complications and mortality. We reported the present case because of the rarity and potential benefits of the approach, when other approaches, such as temporomandibular subluxation, parotid dissection, division of the digastric muscle, and resection of the styloid process, are not expected to provide the needed accessibility.

The incidence of postoperative complications related to carotid body resection has been relatively high, although the incidence has decreased during the past two decades. The complications have mainly included cranial nerve injury, stroke, and hemorrhage. Nerve injuries mainly affect the hypoglossal nerve, the vagus nerve and its branches, and the mandibular branch of the facial nerve. The risk of mandibulotomy includes inferior alveolar nerve anesthesia, tooth loss, and malocclusion, malunion, or nonunion of the mandible and might warrant tracheostomy.^4^^,^^10^ Our patient had experienced several complications; however, the complications had not resulted from the mandibulotomy itself but from the large size and invasiveness of the tumor and thrombosis of the conduit used to reconstruct the resected ipsilateral ICA, which had had a very diseased and small-diameter distal carotid artery stump.

## Conclusions

Surgical excision is the treatment of choice for large CBTs. Also, although several surgical approaches are available, midline mandibulotomy will be the most feasible approach for some inaccessible tumors. Diagnosing CBT at an early stage will minimize the risk of unsuccessful excision attempts and associated complications of surgical removal with good postoperative outcomes.
